# How to Verify Non-Presence—The Challenge of Axenic Algae Cultivation

**DOI:** 10.3390/cells11162594

**Published:** 2022-08-20

**Authors:** Leo Pokorny, Bela Hausmann, Petra Pjevac, Michael Schagerl

**Affiliations:** 1 Department of Functional and Evolutionary Ecology, University of Vienna, Djerassiplatz 1, A-1030 Vienna, Austria; 2 Joint Microbiome Facility of the Medical University of Vienna and the University of Vienna, Djerassiplatz 1, A-1030 Vienna, Austria; 3 Department of Laboratory Medicine, Medical University of Vienna, Währinger Gürtel 18-20, A-1090 Vienna, Austria; 4 Department of Microbiology and Ecosystem Science, Centre for Microbiology and Environmental Systems Science, University of Vienna, Djerassiplatz 1, A-1030 Vienna, Austria

**Keywords:** axenicity verification, microalgae, LB-agar-plate test, epifluorescence microscopy, flow cytometry, 16S rRNA gene amplicon sequencing, purification

## Abstract

Many phycological applications require the growth and maintenance of pure algae cultures. In some research areas, such as biochemistry and physiology, axenic growth is essential to avoid misinterpretations caused by contaminants. Nonetheless, axenicity—defined as the state of only a single strain being present, free of any other organism—needs to be verified. We compare the available methods to assess axenicity. We first purified unialgal *Limnospira fusiformis* cultures with an established series of axenicity treatments, and by including two additional treatment steps. The presumable axenic cultures were then tested for their axenic state by applying conventional tests on LB (lysogeny broth) agar-plates, 16S rRNA gene amplicon sequencing, flow-cytometry and epifluorescence microscopy. Only the plate tests indicated axenic conditions. We found a linear relationship between total cell counts of contaminants achieved by flow cytometry and epifluorescence microscopy, with flow cytometry counts being consistently higher. In addition, 16S rRNA gene amplicon sequencing demonstrated its superiority by not only being an efficient tool for axenicity testing, but also for identification of persistent contaminants. Although classic plate tests are still commonly used to verify axenicity, we found the LB-agar-plate technique to be inappropriate. Cultivation-independent methods are highly recommended to test for axenic conditions. A combination of flow-cytometry and 16S rRNA gene amplicon sequencing complement each other and will yield the most reliable result.

## 1. Introduction

Unialgal cultures comprise a single species and usually also heterotrophic bacteria. Algae collections commonly maintain such unialgal cultures [1], which are sufficient or even advantageous over axenic growth for various research questions. Some algae strains are even unable to grow under axenic conditions over longer periods because they are apparently dependent on the presence of heterotrophic bacterial symbionts [2]. Nonetheless, for some research fields, especially in biochemistry and physiology, axenicity is a requirement [3]. The term “axenic” was first introduced by Baker et al. (1942) [4]. They declared that “*Axenic* is derived from two Greek words: *A*—meaning *without* or *free from*, and *Xenos*—denoting a *stranger* or *foreign life*. An axenic organism, as here defined, is a species free from any life apart from that produced by its own protoplasm” [4] (p. 116). According to Brand et al. (2013), an axenic culture is defined as a culture containing no other living organism except the strain of interest [1]. A more pragmatic approach defines an axenic (or pure) algal culture as a culture without any detectable contaminants [5]. Extremely strict definitions include not only living organisms but even viruses [6]. Guillard (2005) stated that “the goal of purification methods is to obtain a viable culture of a single species, free of all other species (“contaminants”) whether eukaryotes, prokaryotes, or viruses” [5] (p. 117).

In the past, various protocols for algae purification have been published. They can be assigned into two general approaches: (1) separating the strain of interest and its contaminants physically and (2) killing all living organisms except the species of interest [5]. In most cases, applying just one purification method is insufficient to achieve axenicity because different contaminants have to be tackled with different approaches. This calls for combining various treatments to generate an axenic culture. The most common method is based on cell dispersion on agar-plates by either streaking or spraying via an atomizer [6]. This isolation technique can be improved by repeated transfers of young colonies to obtain axenic cultures [7]. Other established methods based on the principle of physical separation include micro-manipulation [8], filtration [9], selective gas vesicle collapse [10], cell washing by micro-pipetting [9] and separation by density gradient centrifugation [11]. One of the most advanced methods is fluorescence-activated cell sorting (FACS) [3,12]. Techniques belonging to the killing-approach include lysozyme treatments [13], antibiotic treatments [14,15,16,17], short-term UV irradiation [9] and thermal treatments [18].

Compared to non-extremophiles, growth and maintenance of extremophile organisms is easier to achieve because potential contaminants often do not survive extreme conditions [19]. We therefore focused on the alkaliphilic filamentous cyanoprokaryote *Limnospira fusiformis* (Voronichin) Nowicka-Krawczyk, Mühlsteinová & Hauer [20], which is cultivated worldwide on a large scale and commercially sold under the trade name “Spirulina” [21]. For the purification process, we followed the protocol of Sena et al. (2011) developed for the closely related species *Limnospira maxima* (Setchell & N.L.Gardner) Nowicka-Krawczyk, Mühlsteinová & Hauer [16]. Additionally, we tested two slightly modified variants of their established purification treatments.

The main focus of this study was the assessment of methods to verify axenicity. Axenicity tests can be grouped into four categories. (1) The oldest but still most popular technique is contamination tests on solid (agar-plates) and in liquid media [6]. Enriched media such as LB (lysogeny broth), R2A (Reasoner’s 2A) and potato dextrose agar are still widely used [22]. Basal algal growth media, enriched with organic substances, can be used as well [5]. In liquid media, grey colouring and increasing turbidity are considered as evidence for the growth of contaminants, whereas plate tests mainly focus on screening colony-forming units [6]. (2) Microscopic examination via direct visual approach includes different methods of light microscopy [6], including epifluorescence techniques with staining agents such as 4′,6-diamidino-2-phenylindole (DAPI) [23]. (3) A highly advanced technique to verify axenicity and quantify potential contaminants is flow cytometry (FCM) [24]. (4) Sequence-based methods such as qPCR or high-throughput (amplicon) sequencing are currently the most sensitive methods for axenicity confirmation [25].

This study was initiated by a survey in which we contacted algae culture collections around the world and asked for their method to assess axenicity of cultures. We then tested four approaches for their applicability and informative value: (1) LB-agar-plate tests, (2) epifluorescence microscopy using DAPI staining, (3) FCM using SYBR Green 1 staining and (4) 16S rRNA gene amplicon sequencing. Advantages and disadvantages of the respective method are identified and discussed.

## 2. Methods

### 2.1. Survey

We contacted 58 algae culture collections located around the world and requested information on if and how they verify axenicity of cultures (i.e., which specific methods they use to test for axenic conditions). Interviews were performed in November 2020.

### 2.2. Strain and Long-Term Cultivation Conditions

We used the unialgal, clonal *Limnospira fusiformis* strain ASW 01 100 (Algensammlung Wien, algae culture collection of the University of Vienna, Wien, Austria) with maintenance culture conditions provided, namely 25 °C and a day:night cycle of 12 h:12 h (warm-white fluorescence tubes, 25 µmol photons × m^−2^ × s^−1^) in Zarrouk cultivation medium [26].

### 2.3. Treatments

A dense preculture was raised in Zarrouk medium. Cultivation was conducted in bottles on an orbital shaker (85 rpm) in a greenhouse for two weeks at 26 °C and a light:dark cycle = 16 h:8 h at an intensity of 50 µmol × m^−2^ × s^−1^. Triplicates from the preculture were transferred without any further treatment to serve as controls. If not stated otherwise, cultivation followed preculture conditions.

**Figure 1:**
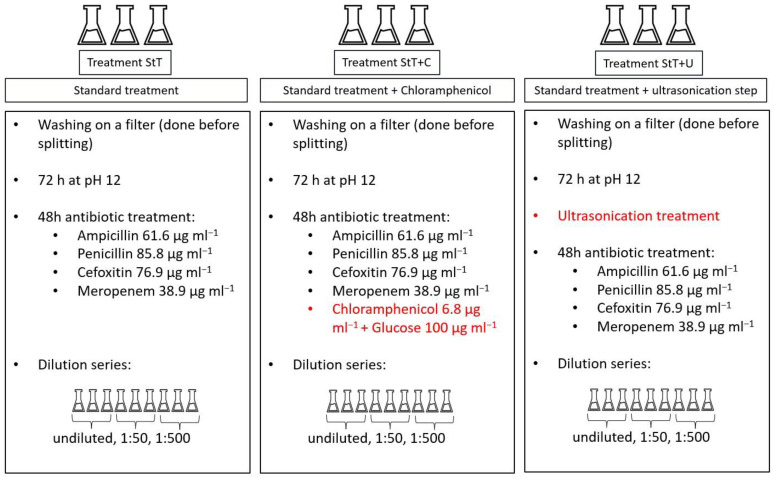
A comparison of the experimental settings of StT, StT+C and StT+U. Differences to StT are marked in red.

The experiment started with a washing step on filters. Algal biomass was put on membrane filters (Isopore hydrophilic polycarbonate, 2 µm pore size) and rinsed with sterile bicarbonate-free Zarrouk medium. The filaments were then transferred into bicarbonate-free Zarrouk medium and split into 9 flasks. Three slightly different treatment approaches were performed, each of them in triplicates (Figure 1).

The standard treatment (= StT) exactly followed the recommendations of Sena et al. (2011) (Figure 1) [16]. First, the rinsed cultures were cultivated at pH 12 for 72 h. The pH was raised by slowly adding 1 mol l^−1^ NaOH. After 72 h, the modified medium was replaced with Zarrouk medium with three centrifugation cycles (each with 2880 rcf for 10 min at 20 °C). After each centrifugation cycle including medium replacement, cultures were briefly vortexed until pellets were dispersed. Subsequently, the cultures were treated with antibiotics for 48 h in the dark. The cocktail contained the following: ampicillin (61.6 µg mL^−1^), penicillin (85.8 µg mL^−1)^, cefoxitin (76.9 µg mL^−1^) and meropenem (38.9 µg mL^−1^). The antibiotic-enriched medium was then again replaced by Zarrouk medium as explained previously. Serial dilutions were performed as follows: From each treated culture, 1 mL was taken and diluted with 49 mL sterile Zarrouk medium (1:50). Then, 5 mL from the homogenized 1:50 diluted culture was taken and diluted with 45 mL sterile Zarrouk medium (1:500). Undiluted and diluted cultures were kept and raised.

Treatment StT+C was performed as in treatment StT, but with addition of chloramphenicol (6.8 µg mL^−1^) and glucose (100 µg mL^−1^) (Figure 1) during antibiotics treatment. Treatment StT+U was performed as in treatment StT, but with the addition of an ultrasonication step between pH- and antibiotic treatments (Figure 1). Each sample was treated with an ultrasonicator (Sonifier 250, Branson, Danbury, USA) for 12 cycles of 10 s each at the lowest intensity. Between each cycle, samples were cooled down for 30 s (bottom half of the Greiner tube immersed in cold water, ~10 °C). After ultrasonication, an additional medium replacement was performed as explained previously.

### 2.4. Harvest, Sample Fixation and Storage

Controls and treated cultures were grown until an optical density of around 2 (measured at 750 nm; U-2000 photometer, Hitachi, Tokyo, Japan; quartz cuvette: 10 mm light path) and in-vivo-chlorophyll a fluorescence around 2.5 (measured at EX 410 nm and EM 670 nm; RF-5301 PC Spectrofluorophotometer, Shimadzu, Kyoto, Japan; quartz cuvette: 10 mm light path), followed by harvesting. This procedure ensured comparable culture densities. Harvested cultures were microscopically checked for monoalgal purity, and the remaining culture was filtrated (3 µm pore size) in order to remove algal filaments. A subsample of the filtrate was immediately used for LB-plate tests. Another part was fixed with formaldehyde (final concentration 2%) and stored at 4 °C for epifluorescence microscopy. Another subsample of the filtrate was fixed with glutardialdehyde (final concentration 0.5%) for 10 min at room temperature, shock frosted in liquid nitrogen for 30 min and stored for FCM at −80 °C. The remaining part of the filtrate was harvested for prospective DNA isolation and 16S rRNA gene amplicon sequencing: 2 mL of the respective sample were centrifuged for 30 min at 21,000 rcf at 4 °C, the supernatant carefully removed and the microcentrifuge tube again filled with 2 mL sample, followed by a second and third step (same conditions as before). The cell pellets obtained from a total of 6 mL filtrate were frozen in liquid nitrogen for 30 min and stored at −80 °C until further processing.

### 2.5. LB-Agar-Plate Tests

Heterotrophic contaminants were screened by inoculation of 100 µL filtrate on LB-agar-plates (NaCl 1%, tryptone 1%, yeast extract 0.5%, agar 1.5%). Plate tests were done in triplicates. Inoculated agar-plates were kept in the dark at room temperature for one month and were inspected for growth at regular intervals.

### 2.6. Cell Count by Epifluorescence Microscopy

Formaldehyde-fixed filtrate was processed within 24 h after harvest. First, 1 mL of the sample was diluted with 10 mL sterile Milli-Q water to ensure a homogeneous distribution during subsequent gentle vacuum filtration on a Whatman (Florham Park, NY, USA) Cyclopore track etched membrane filter (0.2 µm pore size). A cellulose nitrate filter (0.8 µm pore size) served as support filter; a hand pump was used to keep vacuum at a maximum of −20 kPa. The membrane filters were then air dried for 15 min and stored at −20 °C until further investigation. For bacterial counts, filters were thawed, mounted on a slide, and the ready-to-use VECTASHIELD mounting medium with DAPI H-1200 (Newark, USA) was applied for staining (approximately 25 µL per slide). After putting a cover slip onto the filter, random fields of view were counted with a minimum of 100 cells per filter using a Zeiss AXIO Imager M1 Epifluorescence microscope (Zeiss EC Plan-Neofluar 100×/1.3 oil objective, Oberkochen, Germany; at least 10 squares per filter). Cell number was then extrapolated to the total filter area.

### 2.7. FCM Analysis

Prokaryotic contamination was quantified using an Amnis CellStream Flow Cytometer (Luminex, Austin, TX, USA) equipped with a 488 nm blue light laser. Samples were thawed and diluted 1+99 with sterile Milli-Q water (except for blanks and pure Zarrouk medium, where dilution was not appropriate). They were then stained with SYBR Green I nucleic acid stain (Invitrogen, Darmstadt, Germany) at a volume ratio of 1:10,000. Samples were incubated in the dark in a heat block at 37 °C for 13 min. The detector channel settings were: side scatter at 100%, forward scatter at 100%, trigger channel laser (488 nm) at 100%. Speed was adjusted to “fast” (14.64 µL min^–1^). Every particle count was recorded. Cell counts were conducted in technical triplicates, and from each measurement four records were taken. A gated area was counted using a plot of the two parameters 488-611/31-C5 versus 488-528/46-C3. Samples featuring an event rate higher than 800 events s^–1^ were diluted 1+199 with sterile MQ, stained, incubated and measured again. Calculations were performed with Amnis CellStream Acquisition and Analysis software (version 1.2.272).

### 2.8. 16S rRNA Gene Amplicon Sequencing

DNA extraction, 16S rRNA gene amplicon sequencing and bioinformatical analysis of the sequencing data were conducted by the Joint Microbiome Facility of the Medical University of Vienna and the University of Vienna (JMF, project ID JMF-2011-B). For DNA extraction, the DNeasy PowerSoil Pro Kit from QIAGEN was used. Thereafter, Illumina MiSeq-based highly multiplexed 16S rRNA gene amplicon sequencing was performed. Target gene (first-step) PCR was used to amplify the V4 regions of the 16S rRNA genes. The following 16S rRNA gene-targeted oligonucleotide primers were used: 515F (GTGYCAGCMGCCGCGGTAA) and 806R (GGACTACNVGGGTWTCTAAT) [27,28]. Barcoding (second-step) PCR with the unique dual barcoding approach (UDB-H12) was applied: two barcoding primers, each consisting of a distinct 12 nt barcode, and one of the two using 16 nt head sequences (5′-BC12_1-H1- 3′ and 5′-BC12_2-H2-3′) were used for amplification. Following this, amplicon normalization, sequencing library preparation, sequencing on an Illumina MiSeq, sequence processing and sequence analysis were performed. All of these standardized workflows, implemented as standard operating procedures (SOPs) for amplicon sequence generation and analysis at the JMF, were performed as detailed in [29]. Amplicon sequence variants (ASVs) were inferred using the DADA2 R package applying the recommended workflow [30,31]. FASTQ reads 1 and 2 were trimmed at 220 nt and 150 nt with allowed expected errors of 2, respectively. ASV sequences were subsequently classified using SINA version 1.6.1 and the SILVA database SSU Ref NR 99 release 138.1 using default parameters [32,33]. Data were submitted to NCBI’s Sequence Read Archive (SRA). The BioProject accession number is PRJNA866304. 

### 2.9. Statistics

Statistical analyses were performed with IBM SPSS Statistics (version 28.0.0.0; Armonk, NY, USA) to compare the efficiencies of StT, StT+C and StT+U. Only data from 1:500 diluted, treated cultures (considered as our “end products”) and controls were taken into account for analysis. An ANOVA and a Bonferroni post hoc test were performed for FCM data (cells × mL^−1^) to investigate whether differences in total cell counts were significant (total *n* = 12; control *n* = 3, StT *n* = 3, StT+C *n* = 3, StT+U *n* = 3). An ANOVA and a Bonferroni post hoc test were performed for taxa richness data (numbers of detected ASVs) to investigate whether differences in contaminants’ taxa richness are significant (total *n* = 12; control *n* = 3, StT *n* = 3, StT+C *n* = 3, StT+U *n* = 3).

## 3. Results

From 58 algae culture collections, 21 responded to our inquiry. Six collections do not maintain axenic strains, and two collections did not answer the specific question but explained methods for the removal of contaminants instead. The remaining 13 answers are shown in Table 1.

According to the LB-agar-plate tests, all *L. fusiformis* cultures including the controls were axenic even after one month of incubation. FCM data, microscopical cell counts and sequencing, however, indicated contamination in all cultures including the controls, irrespective of treatment and any dilutions.

**Figure 2:**
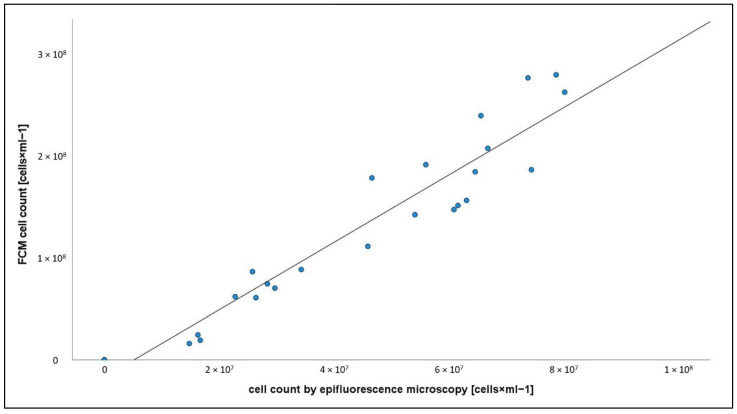
Linear regression of cell count data determined by epifluorescence microscopy and flow cytometry (FCM).

A high and significant linear relationship (R^2^ = 0.916, *n* = 28) between different counting techniques was found (Figure 2). To avoid redundancy, further analyses were based on FCM results.

**Figure 3:**
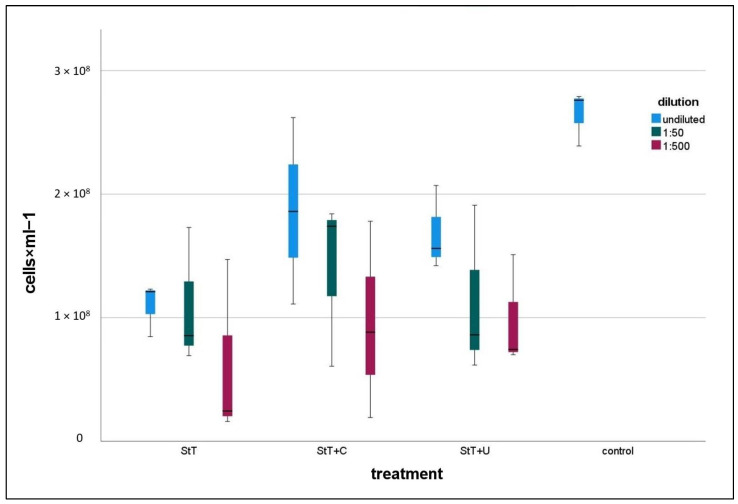
Box plot presenting FCM cell counts of treated and untreated (control) cultures. Treated groups include cell counts of undiluted, 1:50 diluted and 1:500 diluted cultures. StT = Standard Treatment; StT+C = Standard Treatment + Chloramphenicol; StT+U = Standard Treatment + Ultrasonication. *n* for each group = 3.

All treated cultures contained lower numbers of contaminants than the control (Figure 3). In all treatment types, 1:500 diluted cultures were the least contaminated ones. Note here that controls and treated cultures including dilution series were harvested at a comparable optical density, which means different harvest times but comparable growth stage. A statistical comparison of controls and 1:500 diluted, treated cultures revealed that StT and StT+C contained significantly fewer contaminants than controls, whereas StT+U featured no significant differences regarding total cell counts, neither to controls nor StT or StT+C (Table 2).

**Figure 4:**
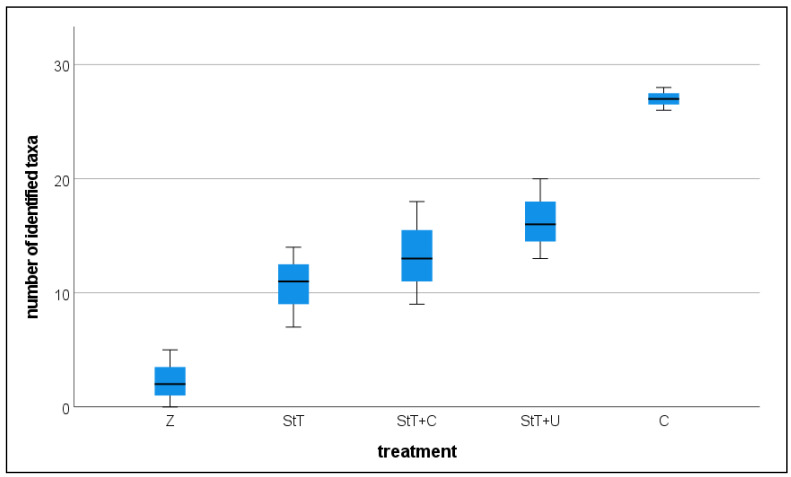
Box plot presenting taxa richness in pure Zarrouk medium (Z), untreated cultures (C) and 1:500 dilutions of treated cultures: Standard Treatment (StT), Standard Treatment + Chloramphenicol (StT+C), Standard Treatment + Ultrasonication (StT+U). *n* for each group = 3.

Sequence data revealed the same pattern. We compared the total count of identified ASVs in each group (1:500 dil. StT, 1:500 dil. StT+U, 1:500 dil. StT+C, control, pure Zarrouk medium; Figure 4) and found a significantly lower taxa richness in the 1:500 dilutions of StT, StT+U and StT+C than in the control (Table 3). No statistically significant difference was found between the three treatments.

**Figure 5:**
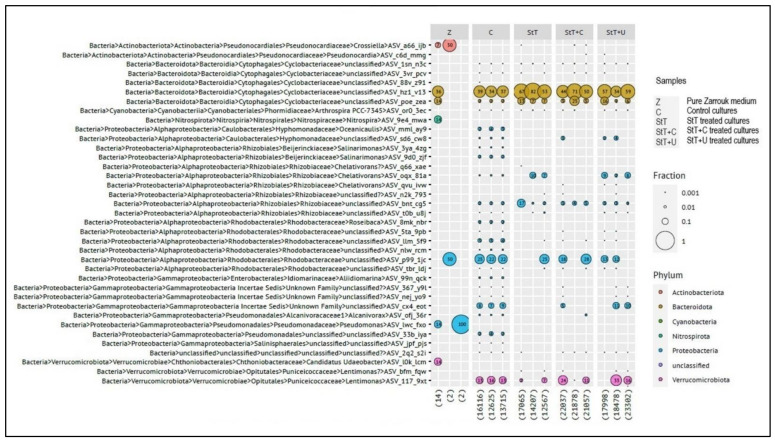
Numbers in bubbles are percentages when more abundant than 1%. Numbers in parentheses are total read counts (depth) per library (i.e., per sample). The diameter of a bubble corresponds with the fraction of reads per library. Fractions shown for higher taxonomic ranks are exclusive of the fractions for separately shown lower taxonomic ranks. The colour within a bubble indicates the phylum an amplicon sequence variant (ASV) belongs to. Solid bubbles are single ASVs. Faded bubbles are the sum of multiple ASVs at different taxonomic ranks.

Contaminants were identified by 16S rRNA gene amplicon sequencing. Most identified contaminants belong to gram-negative taxa. Two taxa belonging to the phylum Actinobacteria constitute an exception, but their read count was extremely low. An ASV affiliated to an uncultivated taxon in the family *Cyclobacteriaceae* (phylum Bacteroidota; ASV_hz1_v13) was the dominant contaminant in controls and all treated cultures (including all dilutions, Figure 5). 

## 4. Discussion

In order to compare and assess techniques for the verification of axenicity, an assumable axenic culture is required. A contaminated *Limnospira fusiformis* culture was chosen and treated. We followed an established purifying protocol by Sena et al. (2011) [16]. The treatment involves a rinsing step followed by pH 12 treatment, the use of four different β-lactam antibiotics and a dilution series. We added two modifications to improve the probability to achieve axenic growth. (1) We applied ultrasonication to minimize contamination by heterotrophic bacteria attached to the algal filament’s surface or embedded in the EPS-matrix (Extracellular polymeric substance) of algal aggregates. Ultrasonication to remove contaminants from cell walls has already been successfully applied in diatoms [34]. An ultrasonication treatment before using antibiotics was also recommended by Vázquez-Martínez et al. (2004) for generating axenic strains of the filamentous cyanobacterium *Phormidium* [17]. (2) In addition to ampicillin, penicillin, cefoxitin and meropenem, the antibiotic chloramphenicol as well as glucose were added. Bacteria surviving antibiotic treatments are usually cells in a stationary phase [35]. This dormant state is not affected by antibiotics, which rely on active growth [36]. Adding glucose as a readily bioavailable carbohydrate induces dormant bacteria to enter active growth, thus increasing the efficiency of the antibiotics. Adding glucose to stimulate bacterial growth and antibiotic susceptibility while treating *Limnospira* cultures was already applied successfully by Choi et al. (2008) [14]. Chloramphenicol as a potent inhibitor of protein synthesis [37] was chosen to extend the spectrum of antibiotics, otherwise maintaining only β-lactam antibiotics. After growing the assumable axenic cultures until a certain optical density, cultures were harvested and we could finally proceed with axenicity testing.

Five out of 13 culture collections that maintain axenic cultures are testing axenicity exclusively by cultivation-based approaches and rely on a single test medium. According to our results, the use of just the single test medium LB-agar does not provide a reliable assessment of the purity-state of our culture. Only a single algae culture collection mentioned that FCM is used for monitoring axenicity. Another culture collection reported that they do not maintain axenic cultures but that they regularly use FCM to check the abundance of contaminants. High-throughput sequencing or qPCR are still not used as purity-tests by any of the culture collections responding to our survey.

Our tests for axenicity revealed contradictory results. LB-agar-plate tests indicated axenic conditions. FCM data, microscopical examinations and 16S rRNA gene amplicon sequencing, however, showed residual contamination after treatments, although with a significantly lower taxa richness compared to the control. Furthermore, StT and StT+C treated cultures contained significantly fewer contaminating cells. The method comparison revealed that, at least for *L. fusiformis* cultures, the classic LB-agar-plate test is an inappropriate method to verify axenicity. This result is not surprising because most microorganisms, in particular oligotrophs, do not readily grow on agar plates. This phenomenon was already described 40 years back as the “great plate anomaly” [38]. Nonetheless, axenicity verification solely using LB-agar-plate tests is still common in many algae culture collections and is even present in recent publications [3].

We compared automated FCM counting (stain SYBR Green 1) and manual epifluorescence counts (DAPI). In agreement with other studies [39,40], we found a very high correlation of the counts, although FCM consistently reported higher particle numbers. Vu et al. (2018) stated that DAPI and SYBR Green 1 unselectively penetrate and stain the DNA of both living and dead cells [6]. With this in mind, both counting methods used in this study bear the risk of overestimating the number of living cells. The precision of FCM counts is assumed to be 10 times higher than that of epifluorescence microscopy, and sample processing time is reduced by even more than tenfold [41]. Accordingly, FCM is the recommended technique for quantifying contaminants because the result is statistically much more reliable. Problems may occur when bacteria form filaments or aggregates. Nevertheless, bacterial quantification and biomass estimation by FCM is even used for samples from activated sludge, but with physical or chemical disaggregation before counting [42]. Even the valid criticism that SYBR Green 1 stains both living and dead cells could be addressed by additionally using live/dead staining [6].

Furthermore, 16S rRNA gene amplicon sequencing demonstrated its superiority as an efficient tool for axenicity-verification and for identification of persistent contaminants. A strategy combining FCM and 16S rRNA gene amplicon sequencing is a solid way to double-verify axenicity. Simultaneously to the axenicity-check, contaminants will be quantified and identified in cases where axenicity was not achieved. Our study identified a member of the family *Cyclobacteriaceae* as the most abundant contaminant. Based on this information, future approaches might include modified treatments to tackle this dominant contaminant in our culture. The limitation of 16S rRNA amplicon gene sequencing for microbial community analyses is the inability to distinguish if DNA originated from a living or a dead cell [43], which might result in false-positive findings in axenicity tests. Li et al. recommended the use of RNA-based sequencing instead of DNA-based methods for reliably detecting living cells [43]. The sensitivity of molecular biological approaches is extremely high, and the slightest contamination in post-harvest processes may result into false-positive findings. Thus, the inclusion of appropriate processing controls is key to avoiding false positive results. We even detected ASVs in sterile medium, whereby read counts were low, between 2 and 14 (Figure 5).

Another issue deserving attention for axenicity verification is the duration between treatment and starting point of tests. Sena et al. (2011) monitored cultures microscopically before and immediately after each treatment [16], which bears the risk of simply overlooking contaminants. Numbers might be below the detection limit of the chosen method of axenicity verification. The risk for this bias is especially high if dilution series are included and tests are conducted before reaching a high algal density. Test duration is another issue. Choi et al. (2008) published an approach to achieve axenic *Limnospira* cultures including washing steps and two different antibiotic treatments, the first one with imipenem and cycloheximide and the second including neomycin and cycloheximide [14]. After each purification step, subsamples were stained with DAPI and observed microscopically. After the last treatment, the established presumptive axenic cultures were grown in SOT medium with 0.1% glucose for 1 day at 30 °C. Axenicity was tested by inoculation in different test media (1/10 NA, LB and R2A), on both solid agar-plates and in liquid media. Test media were examined by the naked eye and microscopically. Cultures were assessed to be axenic after only two more days without evidence of contamination in any of the test media [14]. For visible colony formation of slowly growing bacteria, this period is certainly too short and might result in incorrect conclusions. Contradictory to the duration (two days) specified in the method chapter, Choi et al. (2008) claimed in the discussion that “Purity verification of axenic *A. platensis* was confirmed for 1 week by test agar plate” [14] (p. 92). Guillard (2005) recommended that “tubes and plates should be examined frequently beginning ca. 48 h after inoculation and left for at least 21 days, or 1 month for cold-water organisms or methylaminotrophs” [5] (p. 129). Sena et al. (2011) evaluated the efficiency of different antibiotic treatments by inoculation on AO agar-plates (glucose 1%, peptone 0.5%, yeast extract 0.3%) but did not specify test duration [16].

Before investing much time and money in generating axenic cultures, the need for axenicity needs to be scrutinized. In most cases, monoalgal cultures will suffice for the research question. In some cases, axenic cultures are not even viable, probably due to the elimination of obligate symbionts [2]. Gao et al. (2020) reported a significantly slower growth for *Microcystis aeruginosa* at axenic conditions compared to cultures with associated heterotrophic bacteria [44]. In order to prove that the growth-promoting effect originates from extracellular substances of the heterotrophic strain B905–1, a cell-free filtrate of strain B905–1 was added to an axenic *Microcystis aeruginosa* culture, resulting in increased growth [44]. Another issue to consider is that each application of antibiotics in the establishment of axenic cultures for scientific or economic usage increases the risk of generating and spreading antibiotic-resistant microbes [6]. Nonetheless, certain fields of research require axenicity. Working with axenic cultures results in less noise and a better sequence coverage in the field of full-length genome assembly enables investigations of microalgae-bacteria interactions without interference by non-target bacteria, and simplifies identification of the producer of a toxin or a valuable bioactive component [6].

The question arises whether it is possible to prove true axenicity. The absence of bacterial growth cannot ensure axenicity because many bacteria do not respond to standard enrichments [2]. No general method of choice exists; all methods have their limits. Even deep sequencing could fail to detect a contaminant [45]. Barsanti & Gualtieri (2005) noticed that “there is no way of demonstrating that a microalgal culture is completely axenic” (p. 212), and therefore axenicity describes a culture condition without demonstrable prokaryotic and eukaryotic contaminants [2]. Pinevich et al. (2018) stated that “an absolutely pure culture belongs to the sphere of abstract theory: in practice, ‘pure culture’ refers to a certain probability level” [45] (p. 1517). If no contaminants could be detected, you can conclude only that “according to your test methods, your culture is axenic” [46] (p. 2).

## 5. Conclusions

If axenicity is required, reliable assessment tools are obligatory. It is mandatory to specify the verification methods used to test for axenicity. We highly recommend the combination of FCM and 16S rRNA gene amplicon sequencing for axenicity tests. As these methods might be too time-consuming and too expensive for monitoring purposes in algae culture collections, they can be combined with other tests such as multiple agar-plate tests and microscopy. Routine screening can be performed by the easy-to-go methods and at regular intervals; these tests can then be complemented by FCM and 16S rRNA gene amplicon sequencing.

## Figures and Tables

**Table 1:** Survey results. The table shows how many algae culture collections are using which approach to test for axenicity. Only applicable answers from culture collections that maintain axenic cultures are included.

Used Test Methods	Number of Collections
Single test medium	5
Multiple test media	2
Single test medium + microscopy	2
Multiple test media + microscopy	3
FCM + Multiple test media + microscopy	1

**Table 2:** Statistical comparison (ANOVA and Bonferroni) of control (untreated) cultures and 1:500 diluted, treated cultures regarding FCM cell counts. Numbers in table cells are *p* values. StT = Standard Treatment; StT+C = Standard Treatment + Chloramphenicol; StT+U = Standard Treatment + Ultrasonication. *n* for each group = 3.

ANOVA—Significance between Groups: *p* = 0.013				
Bonferroni Post Hoc Test:				
Treatments	StT	StT+C	StT+U	Control
**StT**	-	1.000	1.000	0.019
**StT+C**	1.000	-	1.000	0.050
**StT+U**	1.000	1.000	-	0.056
**Control**	0.019	0.050	0.056	-

**Table 3:** Statistical comparison (ANOVA and Bonferroni) of control (untreated) cultures and 1:500 diluted, treated cultures regarding taxa richness. Numbers in table cells are *p* values. StT = Standard Treatment; StT+C = Standard Treatment + Chloramphenicol; StT+U = Standard Treatment + Ultrasonication. *n* for each group = 3.

ANOVA—Significance between Groups: *p* = 0.002				
Bonferroni Post Hoc Test:				
Treatments	StT	StT+C	StT+U	Control
**StT**	-	1.000	0.449	0.002
**StT+C**	1.000	-	1.000	0.007
**StT+U**	0.449	1.000	-	0.029
**Control**	0.002	0.007	0.029	-

## Data Availability

Sequencing data presented in this study are available in NCBI’s Sequence Read Archive (SRA). The BioProject accession number is PRJNA866304. Cell count data from FCM and epifluorescence microscopy were submitted to the SRA as metadata and are available via BioProject PRJNA866304 as well.
